# The Flavonoid Isoquercitrin Promotes Neurite Elongation by Reducing RhoA Activity

**DOI:** 10.1371/journal.pone.0049979

**Published:** 2012-11-29

**Authors:** Gemma Palazzolo, Peter Horvath, Marcy Zenobi-Wong

**Affiliations:** 1 Cartilage Engineering+Regeneration Department of Health Sciences and Technology, ETH Zurich, Zurich, Switzerland; 2 Light Microscopy and Screening Centre, ETH Zurich, Zurich, Switzerland; Indiana University School of Medicine, United States of America

## Abstract

**Background:**

Neurite formation and synaptic patterning are fundamental to the development of a functional nervous system. Flavonoids are natural molecules known for having beneficial effects on brain health through diverse molecular pathways. Cytoskeletal changes occurring during neuritogenesis and synapse formation often involve Rho GTPases. Here we hypothesized that the flavonoid isoquercitrin promotes neuronal differentiation through Rho signalling.

**Methodology/Principal Findings:**

We performed time lapse imaging of NG108-15 cells during incubation with/without isoquercitrin. Isoquercitrin stimulated extensive neurites enriched in the synaptic vesicle protein synaptotagmin-1. Neurite extension was augmented by the ROCK inhibitor Y-27632 suggesting an inactivation of RhoA/Rho kinase as the mechanism. To test this, we first measured the dose-dependent effect of isoquercitrin on RhoA activity and found a 47% reduction in RhoA activity at concentrations which induced neurites (≥40 µM). Secondly, we tested the ability of isoquercitrin to rescue the neural phenotype in a model of RhoA-induced neurite retraction and found that 40 µM isoquercitrin added to cultures previously treated with the RhoA activator calpeptin produced significantly more neurite length/cell than calpeptin alone. Finally, we tested the hypothesis that isoquercitrin may affect RhoA localization preventing the translocation to the plasma membrane. Unexpectedly, immunolocalization studies showed that RhoA was present in nuclear compartments of control NG108-cells, but underwent translocation to the cytoplasm upon treatment with isoquercitrin. DNA microarrays and reverse transcription - quantitative PCR (RT-qPCR) revealed differences in global gene expression of Rho GTPase family members. These data taken together indicate that isoquercitrin is a potential stimulator of neuronal differentiation, through multiple Rho GTPase mediated mechanisms.

**Conclusions/Significance:**

As several members of the Rho GTPase family are implicated in human neurological disorders/injuries, our results suggest that isoquercitrin could be used in the treatment of these pathological states through its effect on this family of molecular switches.

## Introduction

Flavonoids are a family of plant-derived molecules with putative anti-inflammatory, anti-oxidant and anti-cancer properties. Bioflavonoids have gained attention in the neuroscience arena where they may enhance long term potentiation associated with learning and memory [1], [2] and improve performance of psychomotor tasks [3]. On the cellular level, these molecules may protect cells from oxidative stress [4] and can induce neurite outgrowth via ERK signalling pathways [5] or Cl^−^ influx into the intracellular space through a Na^+^/K^+^/2Cl cotransporter [6]. Quercetin and its glycoside derivatives represent one of the most commonly found flavonoids in the diet, reaching intake levels of 30–40 mg/day [7]. Among them, isoquercitrin (quercetin-3-β-glucopyranoside) has been found to exert cytoprotective effects due to a decrease in oxidative stress markers, such as levels of ROS, protein carbonylation and lipid peroxidation [8], [9], [10], [11]. In the present study, we demonstrate that isoquercitrin induces the formation of long neural processes in NG108-15 neuroblastoma/glioma cells. As certain flavonoids are known to influence RhoA and Rho kinase [12] and these molecular switches are involved in the cytoskeletal changes of neuritogenesis [13], cell polarization and synapse formation, we hypothesized that the activation state of RhoA as well as the gene expression profile of members of the Rho GTPase family were responsible for the neuro-differentiation of the cell line examined.

## Results

### Isoquercitrin induces neurite elongation in a migration-dependent manner

**Figure 1:**
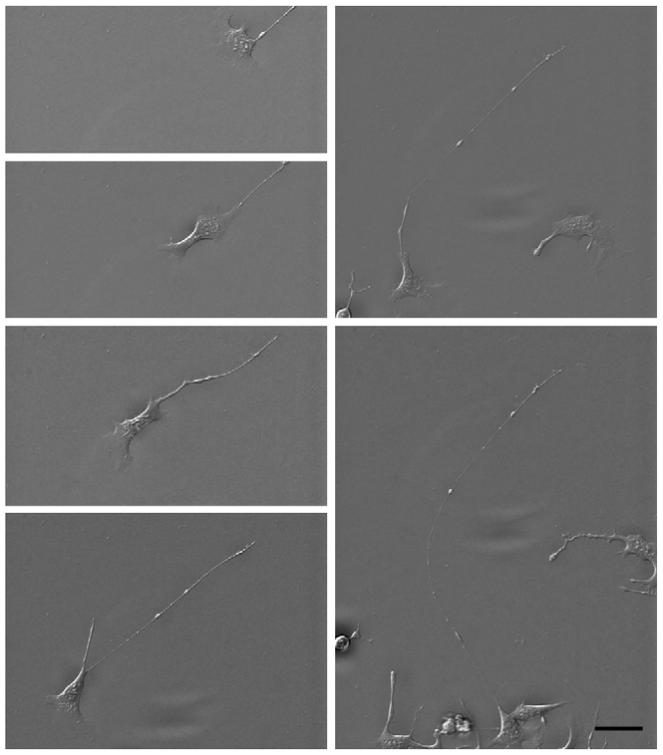
Time lapse acquisition of an isoquercitrin-stimulated NG108-15 cell. The images, acquired every 56 minutes, show the migration path and neurite extension out of the cell body. Note that the neurite tail undergoes a small amount of translation, where the top of each image is at the same position. The video S1 is available in the Supplementary Information. Scale bar = 50 micron.

Incubation of NG108-15 cells with 40 µM isoquercitrin (Q) for 48 hrs showed a progressive elongation of a neurite-like process as the cell underwent migration (Figure 1). In particular, neurites were not formed by extension of a growth cone from a sessile cell, but instead were the result of the neurite being pulled out of the cell as it migrated with its tail still adhered to the substrate (Video S1 in Supplementary Information). Isoquercitrin-induced neurite formation was significantly higher compared to conditions such as DMSO-low serum, serum free medium, and Nerve Growth Factor (NGF) which are reported to induce neural differentiation of neuroblastoma cells [14], [15] (Figure S1). As neurite elongation can depend on cell density [16], we sought to prove that the neurite formation was a direct effect of the drug treatment and not a secondary effect of the cell density. Cells were seeded at four different densities (i.e. 10^3^, 5×10^3^, 10^4^, 2.5×10^4^ cells/cm^2^) and cultured with/without isoquercitrin for 48 hrs. Isoquercitrin induced an elongated phenotype at all cell densities while the carrier-treated control (2 µl/ml DMSO) maintained a non-differentiated morphology even at the lowest cell density (Figure S2).

**Figure 2:**
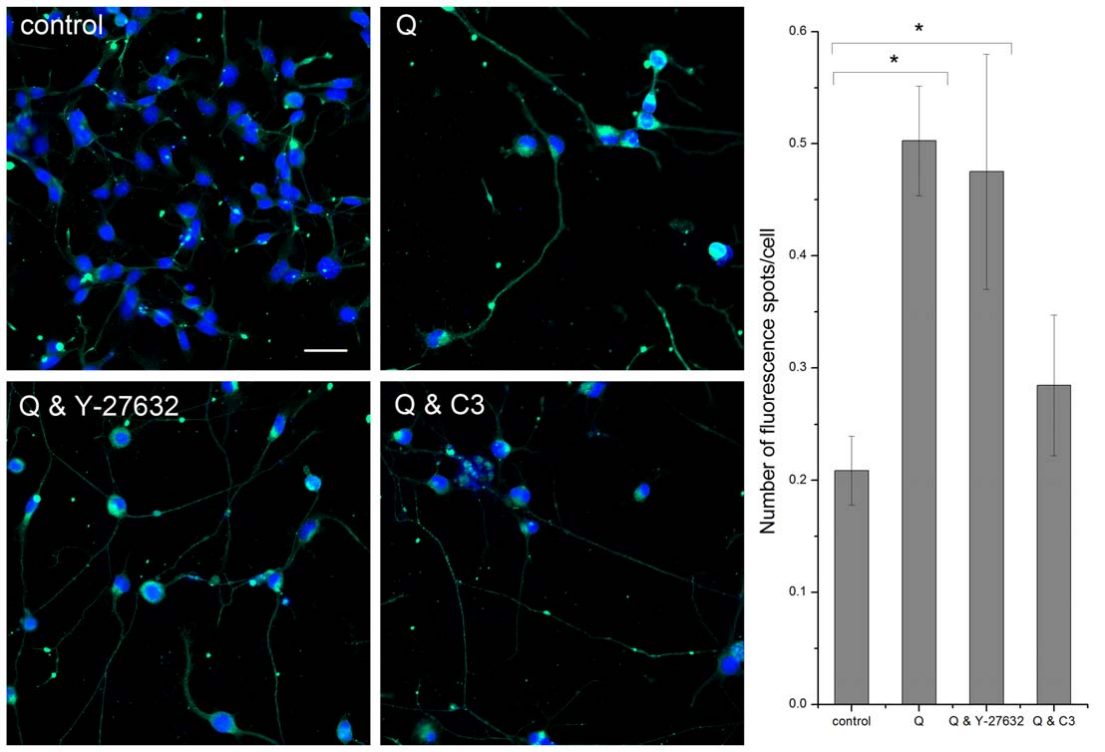
Isoquercitrin and localization of synaptotagmin-1. **Left**, The immuno-staining images show that synaptotagmin-1 localizes especially at spots along the neurites induced by isoquercitrin (Q) alone or combined with Rho inhibitors (Y-27632, C3 transferase). Green = synaptotagmin-1, blue = nuclei. Scale bar = 50 micron. **Right**, The bar graph shows the quantification of the fluorescence spots along the neurites (n = 10 images, one-way ANOVA, * p<0.05). Data are shown as means ± s.e.m.

To test whether the elongation of the neurites might have an effect on synaptic activity, we investigated the effect of isoquercitrin on expression of synapse-associated proteins. Synaptotagmin-1, a calcium sensitive protein involved in synaptic vesicle docking and fusion, is highly expressed in isoquercitrin-induced neurites, as shown in the immunofluorescence images of Figure 2. A similar effect was observed when isoquercitrin was combined with Rho/ROCK inhibitors. Quantitative analysis demonstrated that synaptotagmin-1 localizes to spots along the neurites, implying that isoquercitrin can stimulate a synaptic phenotype.

**Figure 3:**
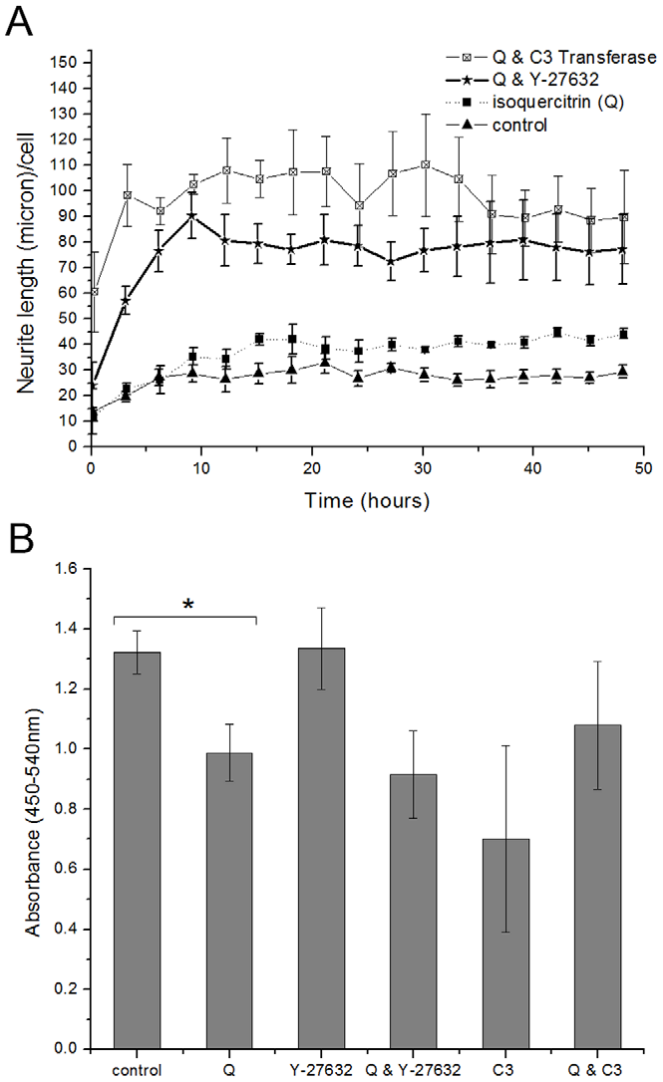
Analysis of neurite length/cell vs. time. **A,** Quantification of neurite length/cell upon addition of isoquercitrin alone or in combination with Rho inhibitors over a period of 48 hours (n = 3, one way ANOVA for repeated measures, F_3,8_ = 17.363, P = 0.001). Acquisition started 30′ after the addition of the molecules. The maximum time for one imaging cycle was 12′. Post hoc Bonferroni test revealed significant differences between the control and the treatments of isoquercitrin combined with each Rho inhibitor, for alpha = 0.05. **B,** Decrease in cell proliferation, due to the presence of isoquercitrin (n = 3, pair-sample t-Test, *p<0.05). Data are shown as means ± s.e.m.

Segmentation of time-lapse images taken during 48 hrs of isoquercitrin incubation with/without Rho inhibitors shows a significant increase in neurite length/cell (Figure 3A). At 48 hrs, isoquercitrin alone caused an increase of 1.5 fold above control (2 µl/ml DMSO). When used in combination with Y-27632, isoquercitrin induced a 3 fold higher neurite length/cell compared to control and a 1.5 fold higher value compared to Y-27632 alone (Figure S3), suggesting a synergistic effect between the two chemicals. When combined with exoenzyme C3 transferase, the flavonoid produced an almost 4 fold increase above control. Nevertheless, C3+isoquercitrin showed a decreased neurite length/cell compared to C3 alone (Figure S3). These data suggest that isoquercitrin may mitigate the C3 action. In fact, C3 induces neurite outgrowth in MN9D dopaminergic cells [17], PC12 cells, and dorsal root ganglion (DRG) neurons, also on inhibitory substrates such as myelin and chondroitin sulphate proteoglycan (CSPG) [18], [19]. To test the effect of the molecules on cell proliferation we performed a BrdU assay after 48 hour cell culture. As it is shown in Figure 3B, isoquercitrin reduced cell proliferation by 25% and was the only substance to have this effect. Specifically Y-27632 did not affect cell proliferation while Y-27632+isoquercitrin induced a decrease of cell proliferation comparable to isoquercitrin alone. This is in agreement with findings from mouse adult neural precursor cells, showing Y-27632 promotes cell process elongation, growth cone-like protrusion formation and cell migration but does not affect cell proliferation [20]. C3 transferase had the lowest value of absorbance, while C3+isoquercitrin showed a value similar to isoquercitrin only. This implies that isoquercitrin can reduce the stronger effect of C3 and supports the above data that showed an attenuation of neurite elongation upon combination of the two drugs.

### Isoquercitrin down-regulates RhoA activity

**Figure 4:**
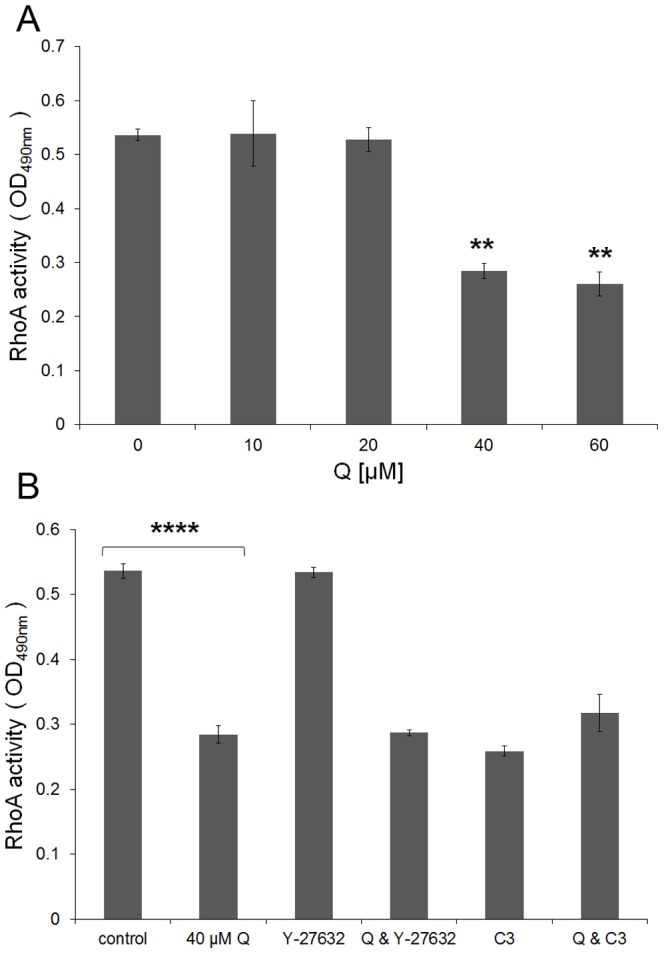
Isoquercitrin inhibits RhoA activity. Cells were maintained for 48 hours in the presence/absence of isoquercitrin and absorbance at OD_490 nm_ was calculated for the cell lysates according to Rho G-Lisa assay. **A,** Isoquercitrin dose dependent graph; **B,** Effects of isoquercitrin on RhoA activity alone and in combination with Rho/ROCK inhibitors (n = 3, one-way ANOVA, ** p<0.01; **** p<0.0001). Data are shown as means ± s.e.m.

We measured the dependence of RhoA activity on isoquercitrin concentration. 10 and 20 µM isoquercitrin did not affect RhoA activity while concentrations of 40 and 60 µM reduced RhoA activity by 47% compared to control (Figure 4A). For all our experiments we have used 40 µM isoquercitrin which was the minimal concentration which induced neurite formation (data not shown). Moreover, we assayed RhoA activity using a combination of isoquercitrin with Rho/ROCK inhibitors (Figure 4B). As expected, isoquercitrin+Y-27632 did not further decrease RhoA activity, because Y-27632 acts downstream in the RhoA signalling cascade.

### Isoquercitrin can rescue the neural phenotype and inhibit RhoA activity

**Figure 5:**
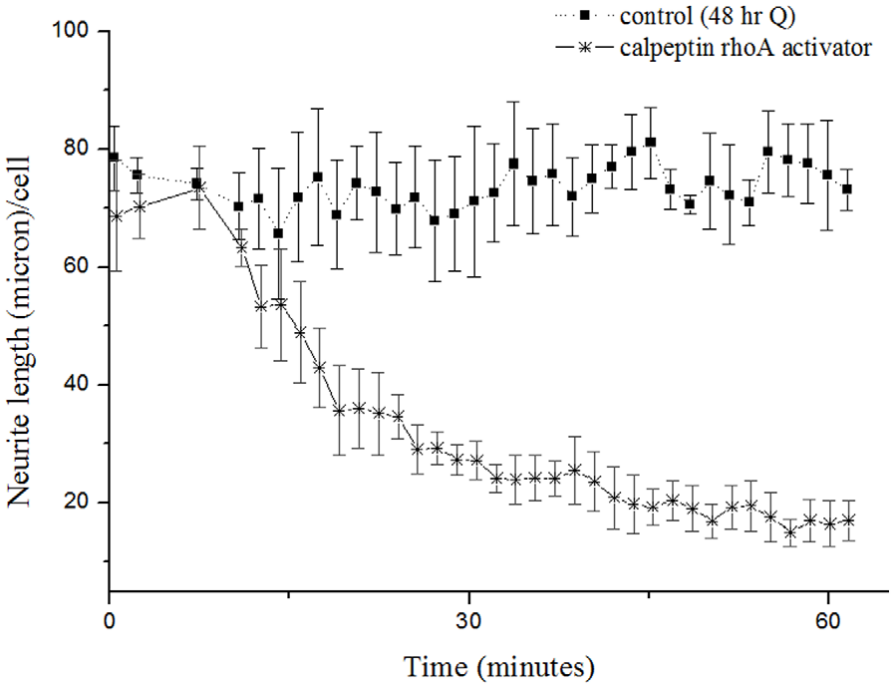
Calpeptin causes a rapid retraction of isoquercitrin induced neurites. After 48 hour incubation with isoquercitrin, neurite retraction was quantified upon addition of RhoA activator calpeptin over a period of 60 minutes (n = 3, one way ANOVA for repeated measures, F_1,4_ = 27.310, P = 0.006). Acquisition was started 5′ after the addition of the molecule. The maximum time of one cycle of acquisition was about 1′. Post hoc Bonferroni test revealed significant differences between the control and the treatment with calpeptin, for alpha = 0.05. Data are shown as means ± s.e.m.

To supplement the above studies, the inhibitory effect of RhoA activation on neurite formation was studied. 48 hour isoquercitrin-conditioned cultures were incubated for 60 minutes with RhoA activator calpeptin (Figure 5). Calpeptin reversed the isoquercitrin-induced phenotype, causing a 77% decrease in neurite length/cell. The majority of neurite retraction occurred within the first 40 minutes (Videos S2, S3, S4, S5, S6, S7 of neurite extension/retraction are available in the Supplementary Information).

**Figure 6:**
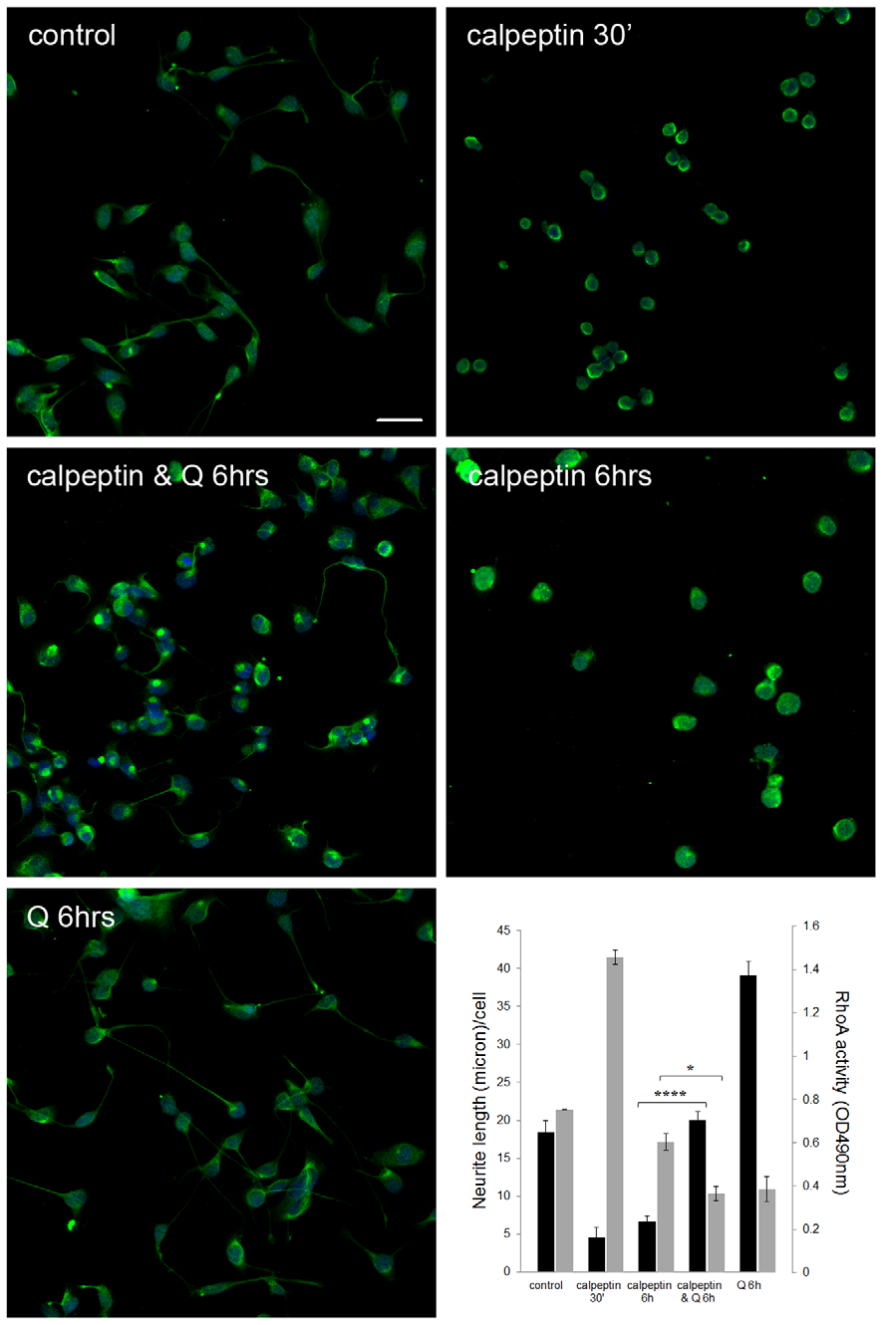
Isoquercitrin is able to restore the neural phenotype in calpeptin stimulated cells. The fluorescence images show 6 hr cultures stained with anti-neurofilament antibody. Green = neurofilament protein, blue = nuclei. Scale bar = 50 micron. **Bottom right**, The double y-axis graph shows: 1) the neurite length (micron)/cell (black bars) measured on the fluorescence images as described in M&M (n = 10 images, one-way ANOVA ****p<0.0001); 2) RhoA activation state (grey bars) evaluated through the Rho G LISA assay (n = 3, one-way ANOVA *p<0.05). Data are shown as means ± s.e.m.

To study the correlation between isoquercitrin-induced neurite elongation and RhoA activity, we tested whether isoquercitrin could restore the neural phenotype which calpeptin had eliminated and at the same time decrease RhoA activity. For this purpose we measured neurite length/cell upon double treatment with calpeptin+isoquercitrin (the latter added 30 min after incubation with calpeptin). As evident in Figure 6, isoquercitrin could reverse, within 6 hours, the neurite suppressive action of calpeptin. In fact, calpeptin+isoquercitrin induced a 3 fold increase in neurite length/cell compared to calpeptin alone. Parallel experiments showed that isoquercitrin-treated cells had a lower RhoA activity (40% reduction) compared to 6 hr calpeptin-conditioned cells, suggesting that neurite length/cell increases as RhoA activity declines. These data indicate an inverse correlation between isoquercitrin-induced neurite elongation and RhoA activity, and strongly suggest that isoquercitrin promotes a neural phenotype by interfering with RhoA activity.

### Isoquercitrin alters RhoA intra-cellular localization

Rho signalling depends on protein localization which can in turn determine its activation state. RhoA is generally found in a GDP-bound inactive state in the cytoplasm. To be active, the protein undergoes post-translational lipidic modification, i.e. geranyl-geranylation, which enables it to interact with the plasma membrane [21]. In order to investigate if isoquercitrin-induced RhoA activity reduction corresponded to a change in protein localization, RhoA immunolocalization was assayed after 48 hr treatment.

**Figure 7:**
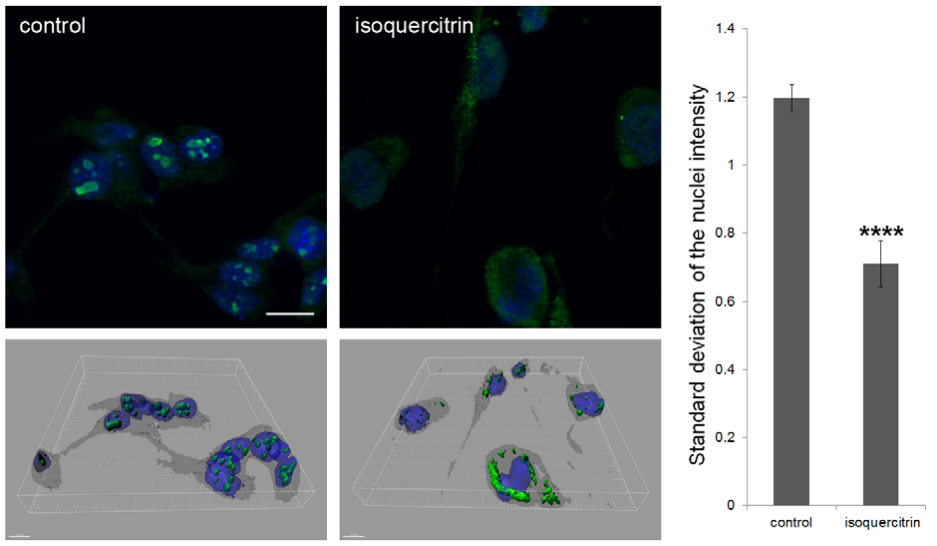
Isoquercitrin causes a change in RhoA localization. **Upper left,** The fluorescent images (acquired with 63× oil objective) show immune-staining of RhoA in 48 hr cultures. Green = RhoA, blue = nuclei. Scale bar = 20 micron. **Bottom left,** 3D projections of z-stack images – obtained with Imaris software - corresponding to 2D images above. Scale bar = 15 micron. **Right,** The bar graph illustrates the standard deviation of the fluorescence in the nuclei normalized by the cytoplasmic fluorescence for each cell. (n = 10 images, pair sample t-Test, ****p<0.0001). Data are shown as means ± s.e.m.

Surprisingly, RhoA was enriched in distinct subnuclear spots of NG108-15 cells while isoquercitrin reduced subnuclear RhoA content, and simultaneously increased the cytoplasmic and sub-membrane counterpart (Figure 7, Videos S9 and S10). The fluorescence ratio of nucleus/cytoplasm was calculated and confirmed that the variation of RhoA staining was higher inside the nucleus of control cells in comparison with the treated ones (1.7 fold). These data indicate that isoquercitrin causes a translocation of RhoA from the nucleus to the cytoplasm.

### Isoquercitrin affects global gene expression

**Figure 8:**
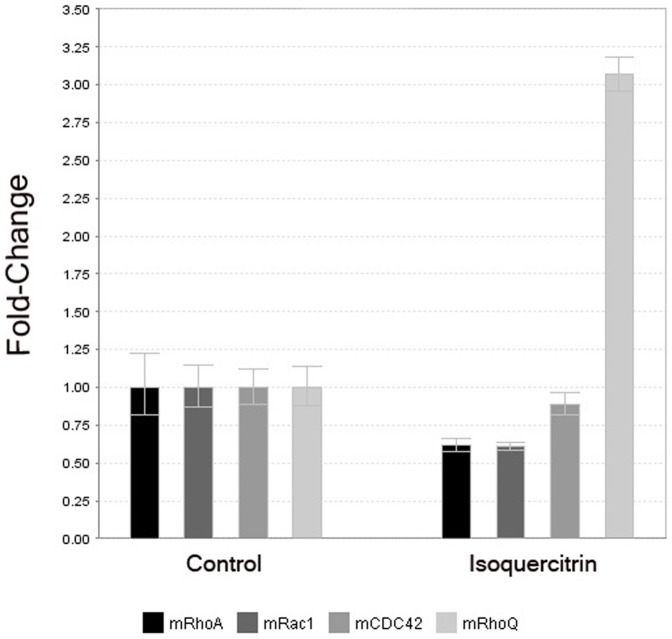
Rho GTPase gene expression is modulated upon 24 hour isoquercitrin stimulation. *rhoA* gene expression is down-regulated, while expression of *rhoQ* (TC10) is upregulated in the isoquercitrin treated cells in comparison with the control.

To screen for additional molecules which could play a potentially important role in inducing the elongated morphology, microarray analysis was performed. We were particularly interested in the Rho GTPase family members, which are implicated in neural migration, cell polarization and synapse formation. Interestingly, isoquercitrin caused a down-regulation of *rhoA* expression by 0.8, in agreement with the reduced protein activity reported above, while the expression of *rhoQ* (TC10) resulted highly upregulated (3.2 fold, p = 0,004). Both results were confirmed by RT-PCR (Figure 8). Other isoquercitrin-associated changes in gene expression included a down-regulation of cell cycle-associated genes and an increase in several proto-cadherins, molecules thought to play a role in synapse formation [22]. We also found an up-regulation of synaptotagmin I (1.9x, p = 0.02), synaptotagmin XI (1.9x, p = 0.01), synaptogyrin (2.1x, p = 0.005) and syntaxin 4A (2.1x, p = 0.001) upon isoquercitrin treatment. The complete microarray data set can be found in the ArrayExpress database and in the Table S1.

## Discussion

The establishment of neuronal connections during development depends firstly on the migration of neurons to their correct physical location [23] and secondly on the guidance of axonal paths to their correct synaptic targets [24]. To our surprise, time-lapsed image acquisition of isoquercitrin-stimulated cells revealed a novel mechanism of neurite formation which was based primarily on migration, not axonal pathfinding. The isoquercitrin-stimulated cells migrated in a manner reminiscent of fibroblast-like “classical” migratory behaviour, namely, beginning with a broad protrusion of lamellipodia and/or filopodia at the leading edge of the cell. In contrast to classical migration, however, the disassembly of focal adhesions at the tail end was delayed, resulting in cells with very long neurite-like processes. Our video analysis revealed instances in which the neurite tail slipped along the substrate, then adhered while the cell moved. The degree of elongation appeared to depend on the efficiency of disassembly of adhesions at the rear of the migrating cell, with both Src and Rho GTPases playing an important role. In particular, RhoA represents a key molecule in the axonal growth inhibitory cascade activated by several factors, e.g. CSPG, oligodendrocyte-myelin glycoprotein (OMgp), myelin-associated glycoprotein (MAG) and Nogo-66 segment from the Nogo-A molecule, which are released by astrocytes and oligodendrocytes at the lesion site upon central nervous system (CNS) or peripheral nerve injury [25]. Targeting the molecules which act upstream or downstream the RhoA-cascade results in extended axonal sprouting [25]. While RhoA activation induces tail release [26] and more rounded cell morphology, the inhibition of its downstream effector ROCK by Y-27632 is associated with an elongated morphology in PC3 human prostatic cancer cells [27], NIH 3T3 fibroblasts [28], and podocytes [29]. In neurons, RhoA activation increased neurite retraction and growth cone release [30], [31] while suppression of p160ROCK induced neuronal morphology in N1E-115 neuroblastoma cells [32]. In PC12 cells, NGF-induced neurite outgrowth was potentiated by the addition of either Y-27632 or Rho inhibitor exoenzyme C3 transferase [33]. Similarly Rho inhibitors induced formation of dendritic processes in melanocytes, while a Rho activator blocked the forskolin induced formation of dendrites [34]. Y-27632 may also stabilize the tubulin cytoskeleton [29] and has been reported to support neuronal survival in injury and neurological disorders [35]. In vivo independent studies have shown that C3 and Y-27632 lead to axonal sprouting upon nervous system damage and improve locomotory activity in spinal cord injury (SCI) models [25]. So far, ibuprofen, the non-steroidal anti-inflammatory drug, represents the best investigated Rho-inhibiting molecule for nervous system repair. In vivo, ibuprofen promotes axonal plasticity/regeneration both in central and peripheral nerve injuries, similarly to C3 transferase and Y-27632 [25].

In the present study we found that the glycoside flavonoid isoquercitrin significantly enhanced neurite formation in NG108-15 cells accompanied by an enrichment of synaptic proteins, i.e. synaptotagmin-1, in spots along the neurites. Isoquercitrin also decreased cell proliferation compared to carrier-treated control cells, implicating that NG108-15 cells resemble more to post-mitotic neurons after stimulation. In rescue experiments we could show that isoquercitrin promoted the neural phenotype in cells which had undergone RhoA activation-induced neurite retraction. Here for the first time we show the correlation between a flavonoid dependent neurite formation and RhoA inhibition which leads likely to a modification of adhesions in the rear of the cell during migration. Therefore we conclude that the isoquercitrin-related neurite elongation is due at least in part to a RhoA/ROCK signalling inhibition.

RhoA, as the other members of the small GTPase family, is activated by interaction with a guanosine exchange factor (GEF) that induces the exchange of GDP with GTP. In order to interact with GEFs, Rho GTPases need to be prenylated. Prenylation (more specifically a geranyl-geranylation) consists of a lipidic chain addition that allows the translocation of Rho GTPase to the plasma membrane enabling then the interaction with the GEF [21]. Ultimately, because RhoA subcellular localization is important in determining activity, we investigated whether the flavonoid could affect its localization. Unexpectedly, we found that in NG108-15 cells RhoA is predominately present inside distinct subnuclear spots and isoquercitrin can reduce this accumulation, thus increasing the relative content of cytoplasmic RhoA. RhoA has been recently found in the nucleus of cancer cells [36], [37] and upon DNA damage [38], suggesting that oxidative stress could cause the translocation of RhoA into the nucleus. Also, the RhoA specific GEF Net1 has been found in the nucleus at a steady state, and ionizing radiation can specifically activate the pool of RhoA in the nucleus through the activation of nuclear Net1 [38]. Recently, Li et al showed that the anticancer drug Taxol induces the translocation of RhoA from the nucleus towards the cytosol and membrane in cancer cells [39]. These studies together with our findings suggest that isoquercitrin could have a dual role in neural differentiation and as a potential anticancer drug.

Our study indicates that the effect of isoquercitrin is not only confined to RhoA. In particular, microarray and real time PCR analyses showed that upon isoquercitrin treatment *rhoA* gene expression is down-regulated, in agreement with the lower RhoA activity; so is *rac1* expression, whose protein product is generally involved in filopodia formation; *cdc42* is less affected. Unlike other Rho GTPases, TC10 is highly upregulated which is of interest, as it has been shown in *in vitro* and *in vivo* studies to be associated with neurite formation [40], [41]. This evidence provides a broader scenario in which isoquercitrin can exert effects on neurite extension through modulating different Rho GTPase signalling pathways. TC10 belongs to Cdc42 subfamily and the two proteins have similar sequences. Although structural and functional similarity between Cdc42 and TC10 has been reported, their expression profile is different. While Cdc42 is ubiquitously present in the tissues, *tc10* gene expression is both temporally and spatially regulated, being mainly expressed during myogenic or neuronal differentiation and following nerve injury [40]. In differentiating hippocampal pyramidal neurons, TC10 activity is crucial for axonal membrane expansion, outgrowth, and establishment of neuronal polarity [42]. In fact, IGF-1 growth factor activates TC10 at the growth cone which in turn recruits Exo70 forming the exocyst complex that is responsible of membrane expansion and also for the axon specification. Interestingly IGF-1 activates TC10 in growth cones isolated from foetal nervous tissue [42]. TC10 expression is found to be high in injured hypoglossal motor neurons as well as in rat dorsal ganglia neurons after axotomy, reflecting a role of TC10 in nerve regeneration [40]. Therefore the up-regulation of TC10 upon isoquercitrin might have a substantial role in the neural differentiation that will need further studies to understand its mechanism of action. Neurite formation results from the cooperative effect of several Rho GTPases and considering only RhoA and TC10 without the other members of this large family and their interactions is a large simplification. The beneficial effect of isoquercitrin is likely the result of a concert of players, which together work to finely regulate differentiation and regeneration.

As a neutriceutical, isoquercitrin can be taken orally and is easily adsorbed by our body where it eventually crosses the blood–brain barrier to reach the retina [43], [44]. As an increasing number of neurological and cancer disorders implicate mutations in the Rho GTPase family of molecular switches [45], the potent influence of flavonoids, including isoquercitrin, on Rho GTPase activity suggests that flavonoids could be beneficial in the treatment of neurological/cancer diseases and for regenerative medicine applications.

## Methods

### Cell culture

The NG108-15 mouse neuroblastoma/rat glioma hybrid cells were seeded at 10^4^ cells/cm^2^ on laminin-coated tissue culture plastic or chambered glass (Lab-Tek™) grown in DMEM containing 10% foetal bovine serum (FBS) and 100units/ml antibiotic/antimycotic. One hour after seeding, 40 µM isoquercitrin (Sigma) alone or in combination with either 10 µM Rho Kinase inhibitor Y-27632 (Sigma) or 0,25 µg/ml Rho inhibitor exoenzyme C3 transferase (Cytoskeleton) was added for 48 hrs. In some experiments, isoquercitrin stimulated cells were incubated with 0,2unit/ml Rho activator calpeptin (Cytoskeleton). For the rescue experiment, 24 hour cultures were first incubated for 30 minutes with calpeptin which causes reversible neurite retraction. Afterwards cells were incubated for 6 hrs with isoquercitrin and the neurite length/cell was measured.

### Live cell imaging

Cells were observed by time-lapse live cell imaging (Cell-IQ, Chip-Man Technologies, Ltd). Three random positions were chosen for each condition and imaged for 48 hours. Quantitative analysis of neurite length/cell was performed using Cell-IQ Analyser Software (Video S8 demonstrates the segmentation and cell counting analysis). In addition, some cultures were imaged at the Light Microscopy Center (www.lmc.ethz.ch) using the Zeiss Life Cell Station equipped with a 20× 0.8NA Plan Apochromat and Metamorph 7.53 software. Acquisition was commenced 1.5 hrs after the addition of isoquercitrin to the cultures and continued for 22 hrs.

### Cell imaging by immunofluorescence

Cells were cultured for 48 hrs on a Chamber Slide™ System (Lab-Tek™) under the above described conditions and then were fixed and permeabilized with 4% formaldehyde +0.1% Triton X100 in PBS for 20 min at 4°C. After blocking with 20%FBS-5%BSA, staining was performed by incubation with the primary antibody for 1 hour at room temperature followed by incubation with the secondary antibody for 30 min at room temperature. Afterwards, cells were incubated with 0.2 µg/ml DAPI for 30 min at 4°C. The imaging was performed using a confocal laser scanning microscope (Carl Zeiss AG). Antibodies: anti-synaptotagmin-1 mouse mAb (1∶200, Synaptic Systems GmbH), anti-neurofilament 200 rabbit mAb (1∶80, Sigma-Aldrich), anti-RhoA mouse mAb (1∶100, Santa Cruz Biotechnology), Alexa Fluor® 488 goat anti-mouse IgG/IgM (1∶400, Life Technologies), Alexa Fluor® 488 goat anti-rabbit IgG (H+L) (1∶400, Life Technologies).

### Segmentation of neurite length

For quantifying the neurite length/cell from immunofluorescence images, the average neurite length/cell was measured through the Ilastik software (an interactive learning and segmentation toolkit) [46]. Cell, neurite and background regions were distinguished. An example is presented in Figure S4A and B. On the segmented images the mean neurite length per cell was measured.

### Cell and synaptotagmin-1 spot detection, spot localization

In case of the fluorescence spot identification, both the detection of spots on the Alexa-Fluor 488 and nuclei on the DAPI channels were performed using an ‘a trous’ wavelet transform-based segmentation method, described in [47]. The proposed method has the advantages of eliminating both shot- and Gaussian-type of noise, detecting objects only in a given radii range with an easy and fast implementation. An example image is shown in Figure S4C.

A machine learning approach was used to automatically decide whether a spot is located on a neurite. First, local properties of the detected spots were calculated to well describe their environment. These properties are the intensity profile around the spots, the number of the intensity profile's local maxima, and the mean intensity of the spots for different widow sizes around it. All together 50 features per spot were calculated. Detected spots were then analyzed with the Advanced Cell Classifier program, which is a machine learning tool for biological images, to classify objects of interest using advanced machine learning methods [48] (Figure S4D and E). This allowed us to determine whether a spot lies on a neurite.

### Nuclei vs. cytoplasm intensity ratio

For the quantification of the RhoA localization, both the DAPI and Alexa-Fluor 488 signals were thresholded. The nuclei were detected as the DAPI objects. The cytoplasms were defined as the difference between Alexa-Fluor 488 and DAPI signal. The measurements were then taken as the ratio between the nuclear and cytoplasmic mean green channel signal and the standard deviation of the intensities.

### Proliferation assay

BrdU label was added to 96-well plate cultures. After 24 hr incubation the BrdU cell proliferation assay was performed according to the manufacturer's instructions (Calbiochem). Absorbance_(450–540 nm)_ was measured with a plate reader (Synergy H1 Hybrid Reader, Biotek).

### RhoA activity assay

For the RhoA activity assay, cells were grown on 6 well plates for the stated time. The RhoA G-LISA™ Activation Assay was carried out according to the manufacturer's protocol (Cytoskeleton, Inc). OD_490 nm_ was read as above.

### Microarray and RT-PCR

Microarray analysis was performed at the Functional Genomics Center Zürich (www.gfcz.ethz.ch). Total cellular RNA from 3 independent cell cultures (+/− isoquercitrin) was isolated using the RNAeasy spin columns with a DNAse digestion step. The purity and concentration were determined using a NanoDrop ND 1000 (NanoDrop Technologies,Delaware, USA) and a Bioanalyzer 2100 (Agilent, Waldbronn, Germany). RNA (600 ng) was reverse transcribed into double-stranded complementary DNA (ds-cDNA) in the presence of RNA poly-A controls, RNA Spike-In Kit, Two-Color. The ds-cDNA was *in vitro* transcribed in the presence of Cy3 (control) and Cy5 (flavonoid-stimulated) labelled nucleotides using a Quick Amp labelling, two-color kit (Agilent P/N 5190-0444, Waldbronn, Germany). Transcription products (1.65 µg) were blocked, fragmented and hybridized to whole Mouse Genome 4x44k OligoMicroarrays (Agilent G4122F) for 17 h at 65°C. Arrays were then washed according to the manufacturer instructions and an Agilent Microarray Scanner used to measure the fluorescent intensity emitted by the labeled target. Total RNA was also reverse transcribed using oligo-dT primer and Superscript III enzyme (Invitrogen). The cDNA sequences for *gapdh* (Fwd: 5′CAT GGC CTT CCG TGT TCC TA3′, Rev: 5′GCG GCA CGT CAG ATC CA3′) as the housekeeping gene, *rhoA* (Fwd: GGG CGT GGA TGC GTT CT, Rev: ACG CGC GCA CAC TCT CA), and *rhoQ* (TC10) (Fwd: GCGCGTCCTGTGGGATT, Rev: GCTCCAAGCGGACATCAGTT) were amplified in triplicate by Real Time RT-PCR, using a StepOnePlus thermocycler with Fast SYBR Green Master Mix (Applied Biosystems).

### Statistical analysis

ANOVA statistical tests were used to estimate global differences among the different treatments and pair sample t-Test was used for comparison between two data sets. Subsequently Bonferroni's post hoc test was applied for multiple comparison, with alpha = 0.05. Statistical analyses were performed with SPSS 19.0 and OriginPro8.

## Supporting Information

Figure S1 **Isoquercitrin promotes an extensive neurite elongation compared to classical differentiation conditions.** We compared the effect of isoquercitrin to other conditions that are reported to induce neural differentiation of neuroblastoma cells, including DMSO-low serum, serum free medium [14] and NGF [15]. Isoquercitrin caused a significant neurite elongation, in comparison with the control as well as with the other conditions which themselves were not significantly different than control. Representative images of neurofilament stained cells. Green = neurofilament, blue = nuclei. Scale bar = 50 micron. **Bottom right,** Bar graph showing the quantitative analysis of neurite length/cell for all the conditions. (n = 10 images; one-way ANOVA, **** p<0.0001). Values are shown as means+s.e.m.
(TIF)

Figure S2 **Isoquercitrin induces neurite formation regardless the cell density.** Representative images of cells which were grown in the presence/absence of isoquercitrin for 48 hrs. **C1, C2, C3, C4** represent the carrier-treated control at 10^3^, 5×10^3^, 10^4^, 2.5×10^4^ cells/cm^2^, respectively. **Q1, Q2, Q3, Q4** represent the isoquercitrin treated samples at the different cell densities, as described above. Scale bar = 50 micron.
(TIF)

Figure S3 **Isoquercitrin and Rho/ROCK inhibitors stimulate neurite elongation.** Representative images of differently treated cells at 48 hrs, acquired with the camera of the Cell-IQ. Scale bar = 50 micron. **Bottom right,** The graph of neurite length/cell vs. time shows all the conditions including Y-27632 and C3 transferase alone.
(TIF)

Figure S4 **Steps of computational analysis.** **A and B,** The original fluorescent image and its segmentation using machine learning-based approach are shown. White: neurite area, light gray: cell, dark gray: background. **C,** An example image with detected nuclei (white crosses) and spots (red crosses). Three sample dots are highlighted: (1) true detection, (2 and 3) false detections, which were than recognized using machine learning methods. **D and E,** Circular (r = 9 pixels) intensity profile around the highlighted spots and their enlarged images.
(TIF)

Table S1 **Microarray data of isoquercitrin effect on global gene expression.** The raw data from the microarray analysis are found also at ArrayExpress (http://www.ebi.ac.uk/arrayexpress/, Accession number: E-MEXP-3418).
(XLSX)

Video S1 **This movie shows neurite extension from the soma of a NG108-15 cell in the presence of isoquercitrin. The acquisition covers a period between the 6^th^ and 9^th^ hour (.mov, 5.76 MB).**
(MOV)

Video S2 **This movie shows the morphology and the proliferation of control NG108-15 cells.** Time lapse imaging started one hour after cell seeding and continued for 48 hours (.mp4, 8.82 MB).
(MP4)

Video S3 **This movie shows the morphology and the proliferation of NG108-15 cells in the presence of 40 µM isoquercitrin.** Upon treatment cells undergo to a neurite extension greater than the control (video 2). Time lapse imaging started one hour after cell seeding and continued for 48 hours (.mp4, 9.49 MB).
(MP4)

Video S4 **This movie shows the morphology and the proliferation of NG108-15 cells in the presence of 10 µM Y-27632.** Upon treatment cells undergo to a neurite extension greater than the control (video 2). Time lapse imaging started one hour after cell seeding and continued for 48 hours (.mp4, 9.42 MB).
(MP4)

Video S5 **This movie shows the morphology and the proliferation of NG108-15 cells in the presence of 40 µM isoquercitrin plus 10 µM Y-27632.** The combined treatment causes increase in the neurite extension in comparison with the control and the single treatments (videos 2, 3 and 4). Time lapse imaging started one hour after cell seeding and continued for 48 hours (.mp4, 9.25 MB).
(MP4)

Video S6 **This movie shows the morphology of NG108-15 cells in the presence of 40 µM isoquercitrin.** Time lapse imaging started after 49 hours of incubation and lasted for 60 minutes (.mov, 2.8 MB).
(MOV)

Video S7 **This movie shows the morphology of NG108-15 cells in the presence of 40 µM isoquercitrin upon RhoA activator calpeptin (0.2 unit/ml) stimulation.** This causes a quick neurite retraction in comparison with the control (Video S6). Time lapse imaging started after 49 hours from seeding and lasted for 60 minutes (.mov, 2.72 MB).
(MOV)

Video S8 **This movie shows the quantification of neurite length as it is measured by the software Cell-IQ.** The red dots indicate the nuclei while the blue lines represent the neurites. The analysis was performed on images taken during 48 hours of incubation with isoquercitrin (.mp4, 8.30 MB).
(MP4)

Video S9 **This movie shows the 3D projection in all dimensions of a z-stack image of NG108-15 cells cultured for 48 hrs as the control, and immunostained for RhoA.** The protein is mainly localized at nuclear spots. Image acquisition was performed using confocal laser scanning microscope (Carl Zeiss, AG). The movie was created with Imaris x64 7.4.0 software (.mp4, 6.97 MB).
(MP4)

Video S10 **This movie shows the 3D projection in all dimensions of a z-stack image of NG108-15 cells grown for 48 hrs in the presence of isoquercitrin, and immunostained for RhoA.** The protein is mainly distributed in the cellular cytoplasm. Image acquisition was performed using confocal laser scanning microscope (Carl Zeiss, AG). The movie was created with Imaris x64 7.4.0 software (.mp4, 7.36 MB).
(MP4)
