# Correction: The Flavonoid Isoquercitrin Promotes Neurite Elongation by Reducing RhoA Activity

**DOI:** 10.1371/annotation/c281cc87-1a2b-4a47-8930-6f6bca672b95

**Published:** 2012-11-30

**Authors:** Gemma Palazzolo, Peter Horvath, Marcy Zenobi-Wong

There are errors in the Funding statement. The correct Funding statement is: This research has been supported by the 3D NeuroN project in the European Union's Seventh Framework Programme, Future and Emerging Technologies (grant agreement n° 296590), a Marie Heim-Vögtlin grant from the Swiss National Science Foundation (PMPDP2_122997 to MZW) and a Swiss National Science Foundation (grant n° IZK0Z2_133865). The funders had no role in study design, data collection and analysis, decision to publish, or preparation of the manuscript. 

