# Supplementary figures and images for: The Flavonoid Isoquercitrin Promotes Neurite Elongation by Reducing RhoA Activity
Source: PLoS One. 2012 Nov 29;7(11):e49979. doi: 10.1371/journal.pone.0049979 (PMC3510166; doi:10.1371/journal.pone.0049979)

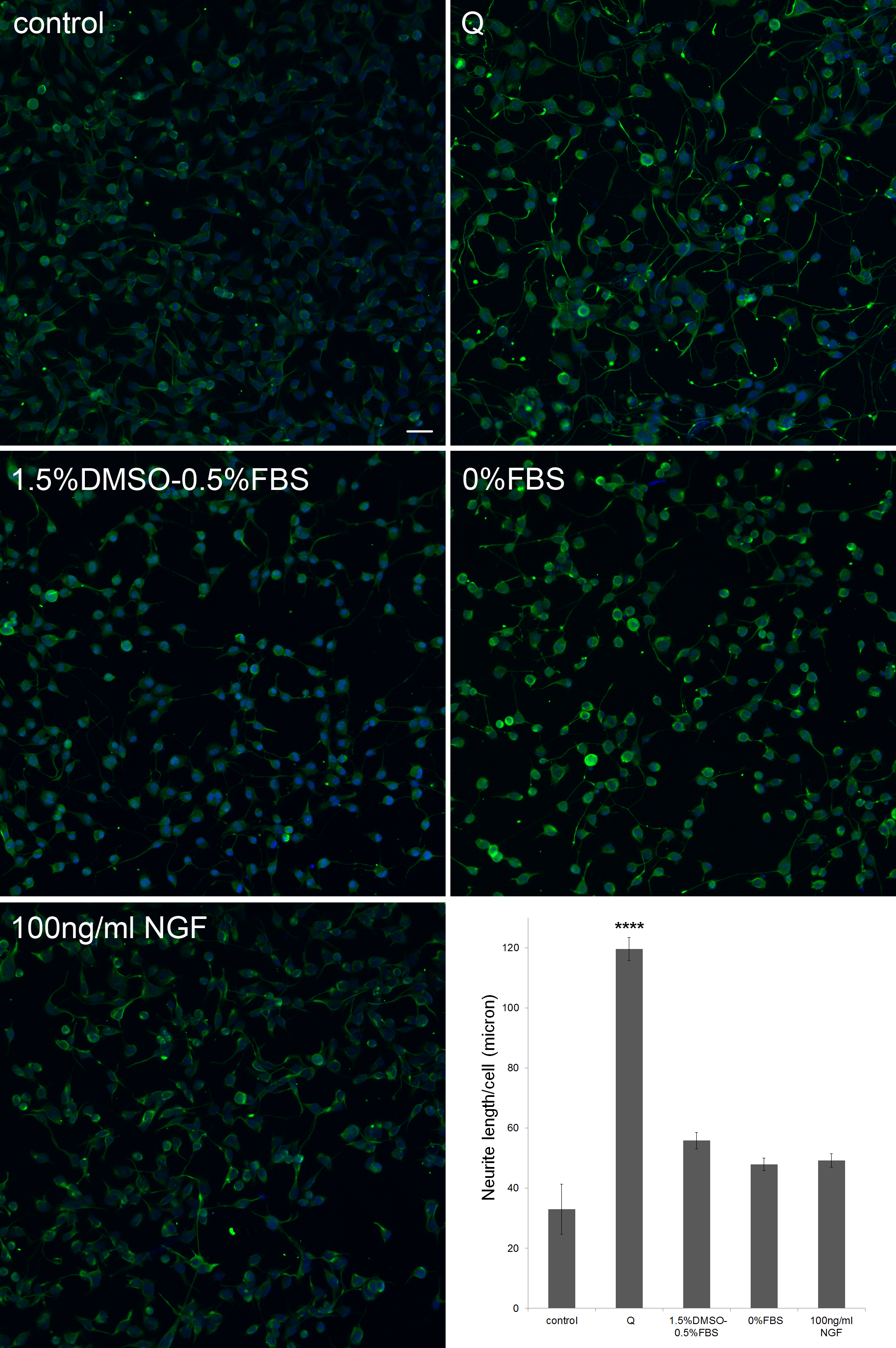

Supplement: Figure S1 — Isoquercitrin promotes an extensive neurite elongation compared to classical differentiation conditions. We compared the effect of isoquercitrin to other conditions that are reported to induce neural differentiation of neuroblastoma cells, including DMSO-low serum, serum free medium [14] and NGF [15]. Isoquercitrin caused a significant neurite elongation, in comparison with the control as well as with the other conditions which themselves were not significantly different than control. Representative images of neurofilament stained cells. Green = neurofilament, blue = nuclei. Scale bar = 50 micron. Bottom right, Bar graph showing the quantitative analysis of neurite length/cell for all the conditions. (n = 10 images; one-way ANOVA, **** p<0.0001). Values are shown as means+s.e.m. (TIF) [file pone.0049979.s001.tif]

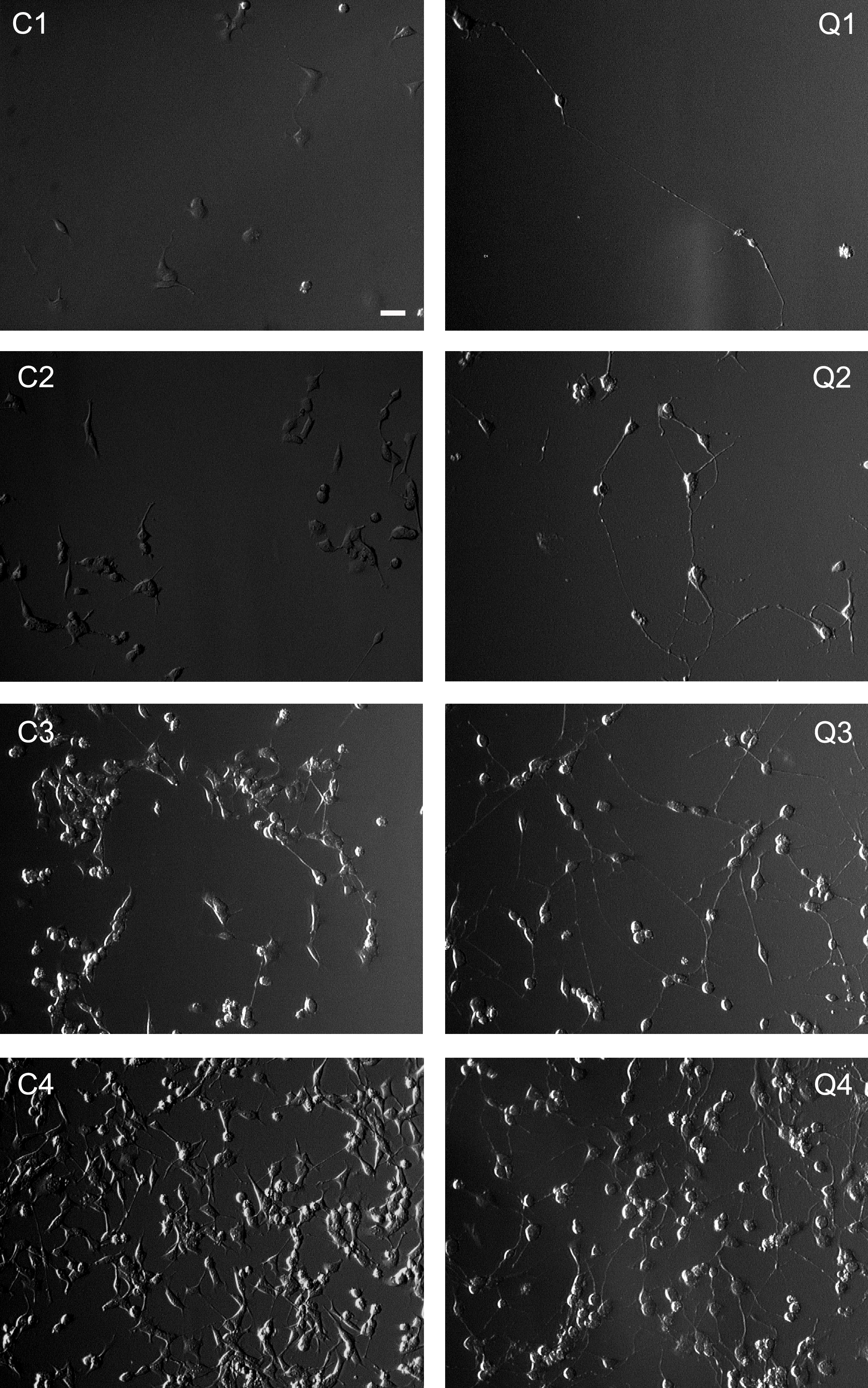

Supplement: Figure S2 — Isoquercitrin induces neurite formation regardless the cell density. Representative images of cells which were grown in the presence/absence of isoquercitrin for 48 hrs. C1, C2, C3, C4 represent the carrier-treated control at 103, 5×10^3^, 104, 2.5×10^4^ cells/cm2, respectively. Q1, Q2, Q3, Q4 represent the isoquercitrin treated samples at the different cell densities, as described above. Scale bar = 50 micron. (TIF) [file pone.0049979.s002.tif]

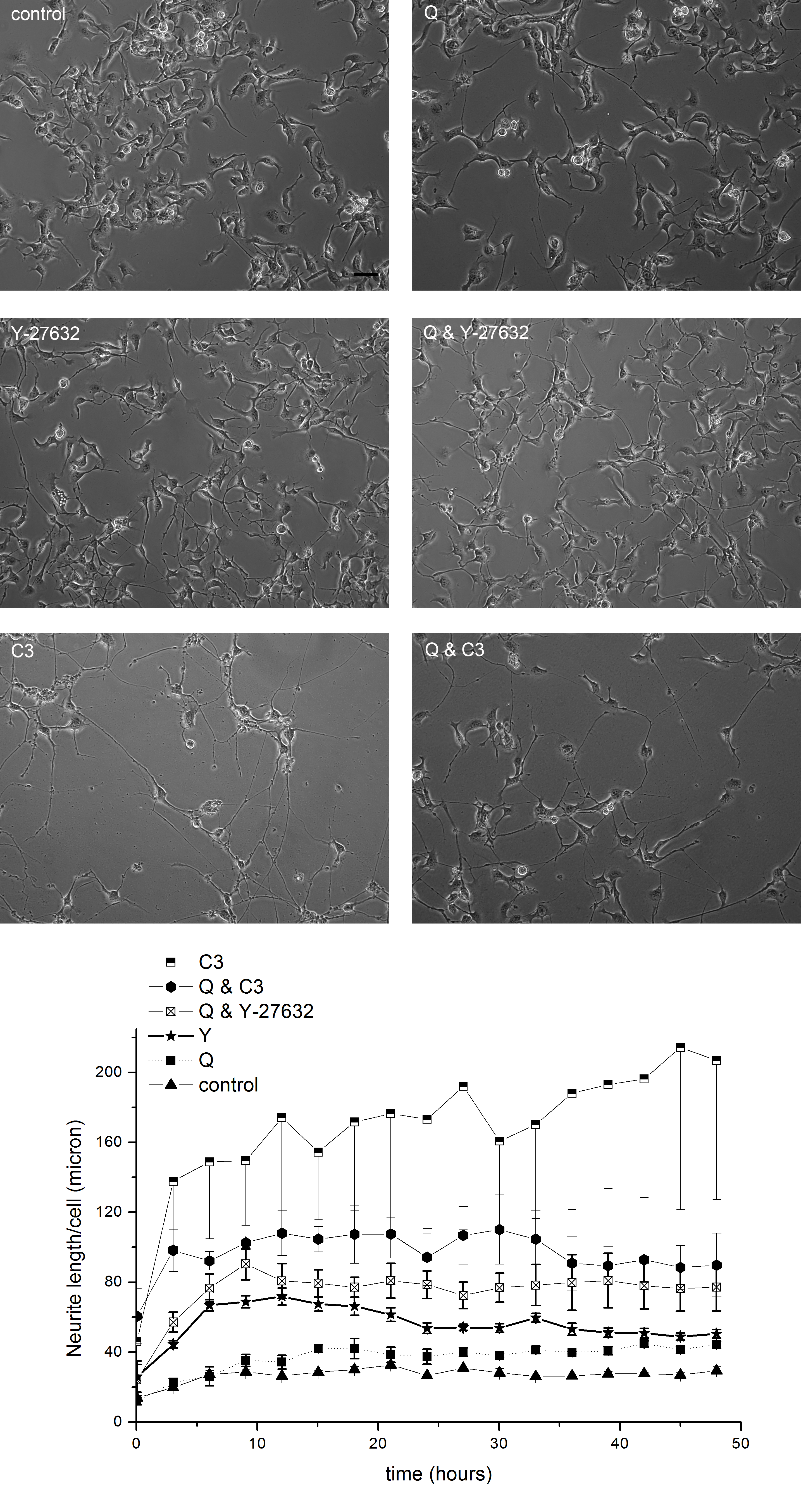

Supplement: Figure S3 — Isoquercitrin and Rho/ROCK inhibitors stimulate neurite elongation. Representative images of differently treated cells at 48 hrs, acquired with the camera of the Cell-IQ. Scale bar = 50 micron. Bottom right, The graph of neurite length/cell vs. time shows all the conditions including Y-27632 and C3 transferase alone. (TIF) [file pone.0049979.s003.tif]

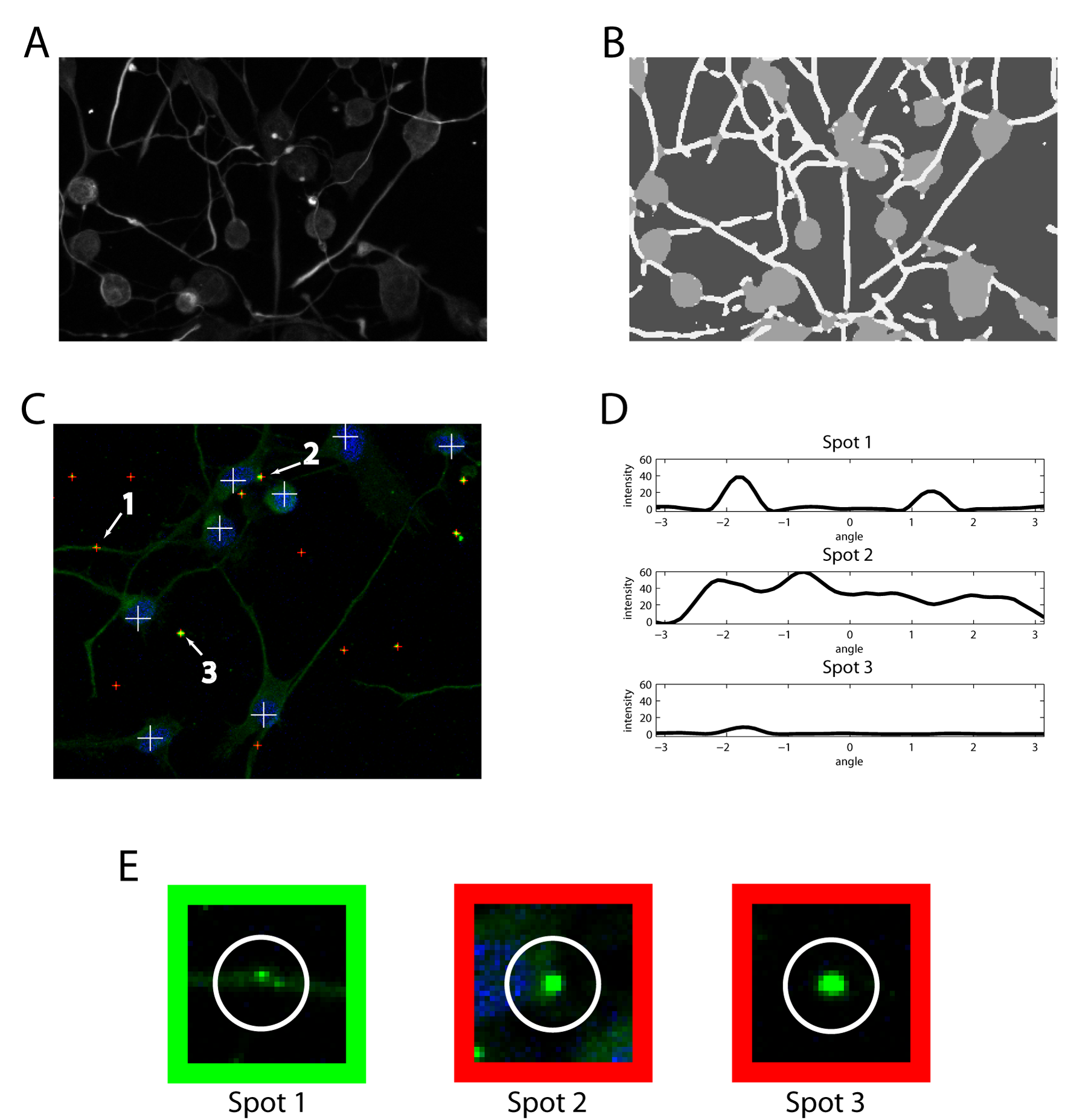

Supplement: Figure S4 — Steps of computational analysis. A and B, The original fluorescent image and its segmentation using machine learning-based approach are shown. White: neurite area, light gray: cell, dark gray: background. C, An example image with detected nuclei (white crosses) and spots (red crosses). Three sample dots are highlighted: (1) true detection, (2 and 3) false detections, which were than recognized using machine learning methods. D and E, Circular (r = 9 pixels) intensity profile around the highlighted spots and their enlarged images. (TIF) [file pone.0049979.s004.tif]
